# Case Report: Effects of Anti-SARS-CoV-2 Convalescent Antibodies Obtained With Double Filtration Plasmapheresis

**DOI:** 10.3389/fimmu.2021.711915

**Published:** 2021-06-30

**Authors:** Diego Curtò, Federica Tomatis, Sara Gastoldi, Miriam Galbusera, Marina Noris, Federico Raimondi, Ferdinando Luca Lorini, Anna Falanga, Marina Marchetti, Giuseppe Remuzzi, Piero Ruggenenti

**Affiliations:** ^1^ Departments of Renal Medicine, Rare Diseases and Molecular Medicine, Istituto di Ricerche Farmacologiche Mario Negri IRCCS, Bergamo, Italy; ^2^ Unit of Nephrology and Dialysis, Azienda Socio-Sanitaria Territoriale (ASST) Papa Giovanni XXIII, Bergamo, Italy; ^3^ Unit of Pulmonary Medicine, ASST Papa Giovanni XXIII, Bergamo, Italy; ^4^ Department of Health Sciences, University of Milan, Milan, Italy; ^5^ Intensive Care Unit, ASST Papa Giovanni XXIII, Bergamo, Italy; ^6^ Immunohematology and Transfusion Medicine, ASST Papa Giovanni XXIII, Bergamo, Italy; ^7^ School of Medicine, University of Milan Bicocca, Milan, Italy

**Keywords:** convalescent antibodies, COVID-19, double filtration plasmapheresis, rituximab, immunosuppression, coagulation biomarkers, hypercoagulability, case report

## Abstract

Passive antibody therapy has been used to treat outbreaks of viral disease, including the ongoing pandemic of severe respiratory acute respiratory syndrome (SARS) coronavirus 2 (SARS-CoV-2) or COVID-19. However, the real benefits of the procedure are unclear. We infused a concentrated solution of neutralizing anti-SARS-CoV-2 antibodies obtained from a convalescent donor with a single session of double filtration plasmapheresis (DFPP) into a 56-year-old woman with long history of unremitting, severe COVID-19. She was unable to establish an adequate antiviral immune response because of previous chemotherapy, including the infusion of the anti-CD20 monoclonal antibody rituximab, administered to treat a diffuse large B-cell lymphoma. The disease promptly recovered despite evidence of no endogenous anti-SARS-CoV-2 antibody production. The observation that passive antibody therapy might prove particularly effective in immunodepressed COVID-19 patients requires evaluation in prospective randomized controlled trial.

## Introduction

“Passive immune therapy,” or “passive antibody therapy,” has been used for more than one century to treat outbreaks of viral disease, especially when specific therapeutic options have been lacking. Starting from the 1918 influenza pandemic and continuing with the more recent pandemics of severe acute respiratory syndrome (SARS), H_1_N_1_ influenza, Middle East Respiratory Syndrome, and Ebola virus disease, this approach has been used also during the dramatic, still ongoing pandemic of SARS coronavirus 2 (SARS-CoV-2) or COVID-19 (1) that, after emerging from Wuhan, China, in 2019, has spread world-wide infecting 154 million and killing 3.2 million people by May 5, 2021 (2). This therapeutic approach relies on the reasonable concept of transferring antibodies from a patient who recently recovered from the disease and developed a robust pathogen-specific humoral response (convalescent antibodies) to another patient who is in the early stages of the disease and has not yet developed (or is unable to develop) an immune response to the infecting agent (3). Transfusion of plasma is historically the first and most frequently used procedure to transfer antibodies from convalescent donors to critically ill recipients. Several uncontrolled studies provided evidence of efficacy of this procedure (4–7). However, a recent prospective, randomized controlled trial found that the use of convalescent plasma did not result in a significant clinical benefit as compared with placebo in patients with severe COVID-19 pneumonia (8). These findings were consistent with results of a randomized controlled trial in patients with moderate COVID-19 that showed no treatment effect against progression to severe disease or death (9). A plausible explanation for these discouraging results could be that the total titer of antibodies provided with plasma was not sufficient to fully neutralize the infecting agent.

The aforementioned limitation could be overcome by providing a larger load of neutralizing antibodies with the use of small concentrated solutions of IgG purified from large amount of convalescent plasma. In this context, the use of selective apheresis methods, such as double-filtration plasmapheresis (DFPP) (10), is a safe and efficient procedure to obtain highly concentrated IgG solutions from convalescent donors. DFPP is a two-step procedure that, after initial separation of donor red blood cells from plasma, allows a further separation of specific molecules from plasma with fractionation filters that retain molecules with a diameter larger than the cutoff diameter of the filter membrane. Specifically, the sieving coefficient for IgG of the Fractionation Filter 2A20 allows to efficiently separate circulating immunoglobulins from other smaller circulating macromolecules (11). With this filter, separated donor IgG are collected and frozen in a sterile collecting bag, whereas plasma along with major part of the albumin and all smaller molecules are merged with red blood cells, and the whole blood, devoid of separated IgG, is re-infused into the donor. Notably, the IgG amount in the collected concentrate is higher as compared with the amount in hyperimmune plasma obtained by simple plasma separation. Moreover, as compared with convalescent plasma, these concentrated and purified IgG solutions could reduce the risk of cardiovascular instability related to fluid overload in critically ill recipient and also the risk of infusional reactions to plasma components different from neutralizing antibodies. Citrate-induced hypocalcemia and bleeding, because of coagulation factor depletion when albumin and saline are used as replacement fluid, are additional donor risks that may complicate the plasmapheresis procedure and can be avoided by DFPP IgG purification.

We successfully used the DFPP procedure to remove nephritogenic anti-PLA_2_R IgG from the circulation of patients with PLA_2_R-related membranous nephropathy (12). These antibodies were collected in a sterile bag and then discarded, whereas plasma and red blood cells were re-infused into the patient. Thus, we reasoned that the same approach could be safely used to purify specific, neutralizing anti-SARS-CoV-2 antibodies from COVID-19 convalescent patients. Rather than discarding these antibodies, we planned to collect, freeze, store, and then slowly thaw and infuse them into critically ill COVID-19 patients to “jump start” their immune system to control the evolution of the disease until a specific immune response could be established (3). Based on this rationale, we used this approach to treat a COVID-19 patient who was unable to establish an adequate antiviral immune response because of previous chemotherapy, including the infusion of the anti-CD20 monoclonal antibody rituximab, administered to treat a diffuse large B-cell lymphoma. This patient recovered from the disease despite evidence that the humoral response to the viral agent was fully sustained by infused donor convalescent immunoglobulins, without any endogenous anti-SARS-CoV-2 antibody production.

## Case Description

**Figure 1:**
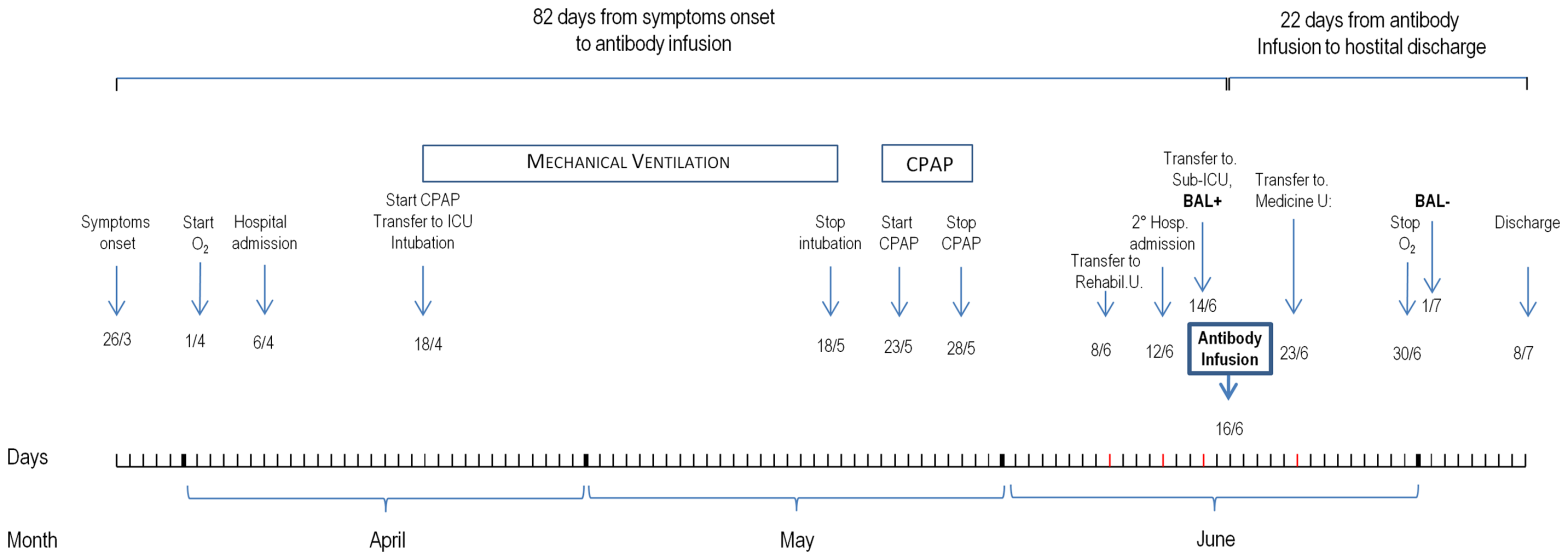
Patient outcome from symptoms onset to hospital discharge.

**Figure 2:**
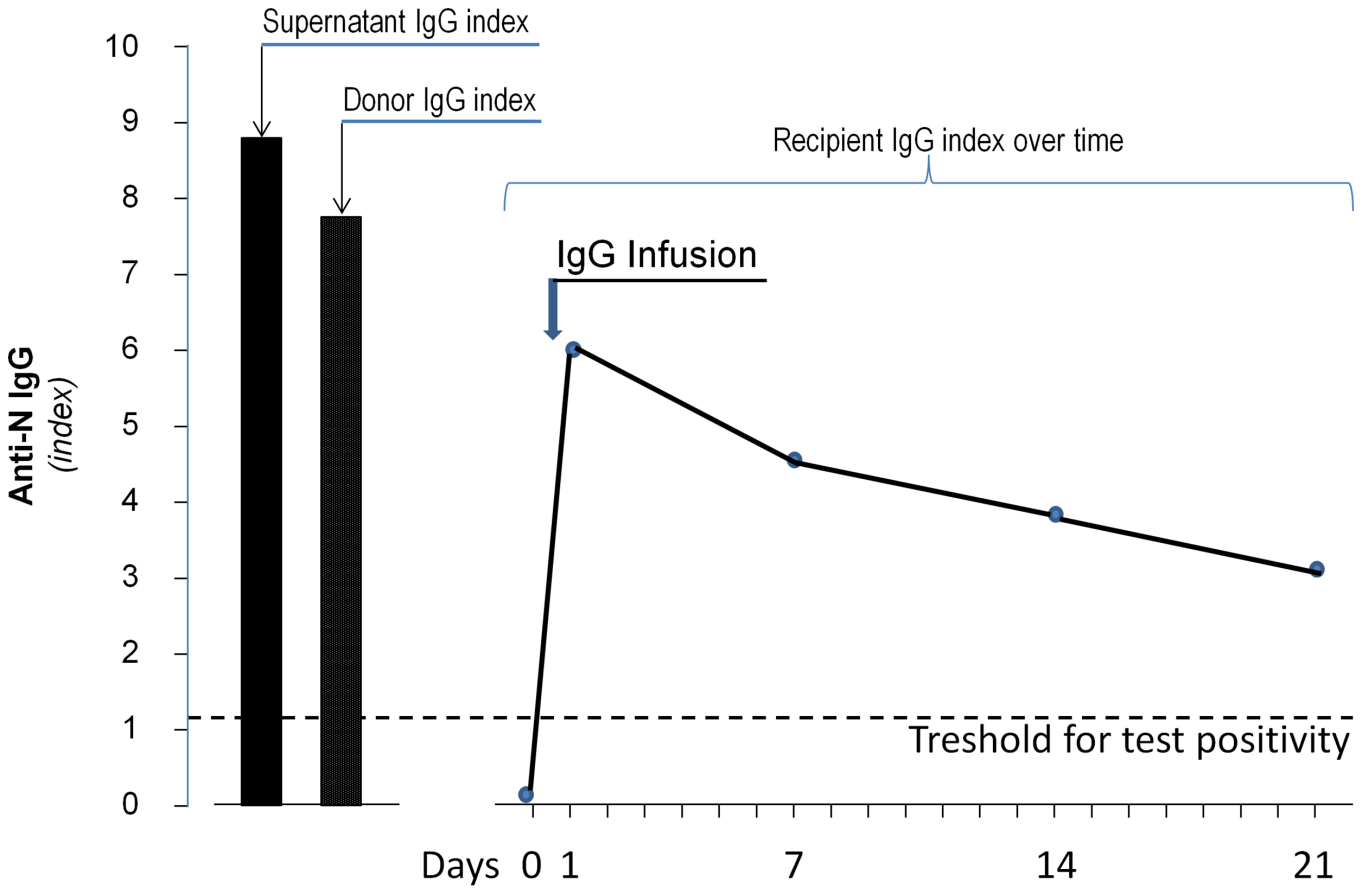
Levels of anti-SARS-CoV-2 N IgG antibodies in the concentrate antibody solution infused to the patient, in antibody donor serum and in recipient sera collected before antibody infusion (day 0) and 7, 14, and 22 days thereafter. The dashed line indicates the threshold for antibody positivity.

**Figure 3:**
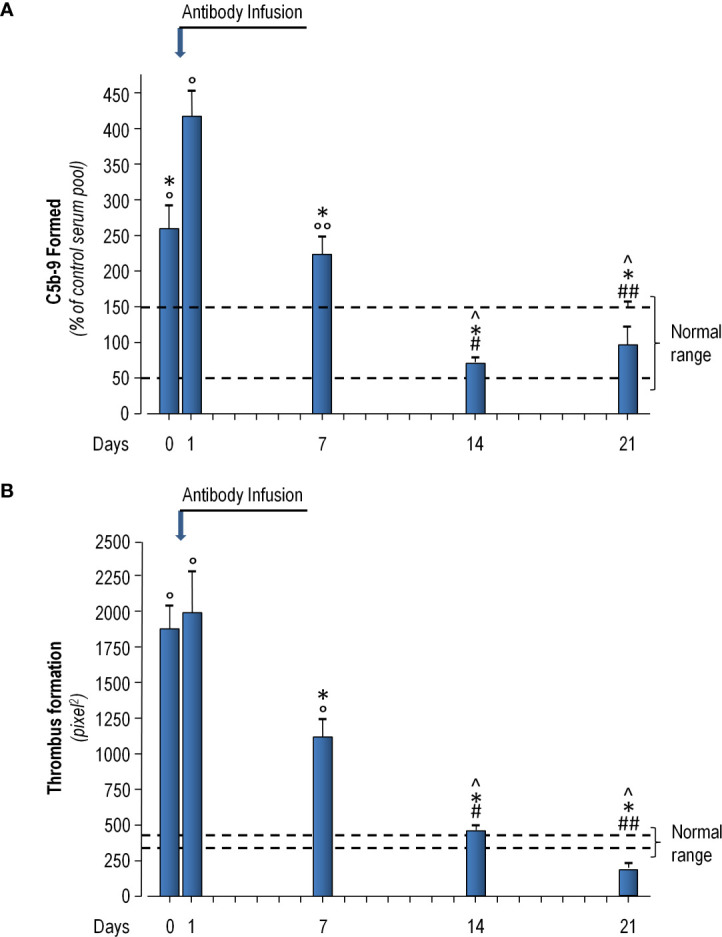
Patient serum-induced ex vivo C5b-9 deposition **(A)** and thrombi formation **(B)** on cultured Human Microvascular Endothelial Cells (HMEC-1) line immediately before antibody infusion (Day 0), and 1, 7, 14, and 21 days thereafter. **(A)** HMEC-1 were incubated for 2 h with serum (diluted 1:2 with test medium, HBSS with 0.5% BSA) from the patient or with a control serum pool. At the end of incubation, cells were washed, fixed, and stained with rabbit anti-human complement C5b-9 complex antibody followed by FITC-conjugated secondary antibody. Fluorescence microscopy was used to view the fluorescent staining on endothelial cell surface, and the HMEC-1 area covered by C5b-9 staining was calculated by automatic edge detection (Image J software) in 15 high power fields. For each sample, the highest and the lowest values were discarded and the mean of the other 13 fields was calculated, and values were expressed as the percentage of C5b-9 deposits induced by a pool of sera from 10 healthy controls run in parallel (reference 100%). Dashed lines indicate upper and lower limit of normal range. **(B)** HMEC-1 were activated with ADP and exposed for 2 h to serum (diluted 1:2 with test medium, HBSS with 0.5% BSA) from the patient or with a control serum pool. Perfusion of heparinized whole blood (heparin 10 U/ml) from an healthy subject (added with the fluorescent dye mepacrine 10 µM, to label platelets) was then performed in a thermostatic flow chamber (37°C) in which one surface of the perfusion channel was a glass slide seeded with a monolayer of endothelial cells at a constant flow rate of 1500 sec^-1^ (60 dynes/cm^2^). After 3 min, perfusion was stopped, and the slide with the endothelial cell monolayer was dehydrated and fixed in acetone for 20 min. Slides were examined under confocal inverted laser microscopy. Fifteen fields for each slide were systematically digitized along the surface and the area covered by thrombi was quantified by Image J (NIH, Bethesda, MD), and expressed as pixel^2^ per field analyzed. For each sample the mean of 15 fields (excluding the lowest and the highest values) was calculated. Dashed lines indicated the area covered by thrombi of control serum pool ± SE. Data are reported as means ± SE. °P<0.0001, °°P<0.001 versus control serum pool; ^P<0.0001 versus day 0; *P<0.0001 versus day 1; ^#^P<0.0001, ^##^P<0.001 versus day 7. Statistical analysis: ANOVA.

**Figure 4:**
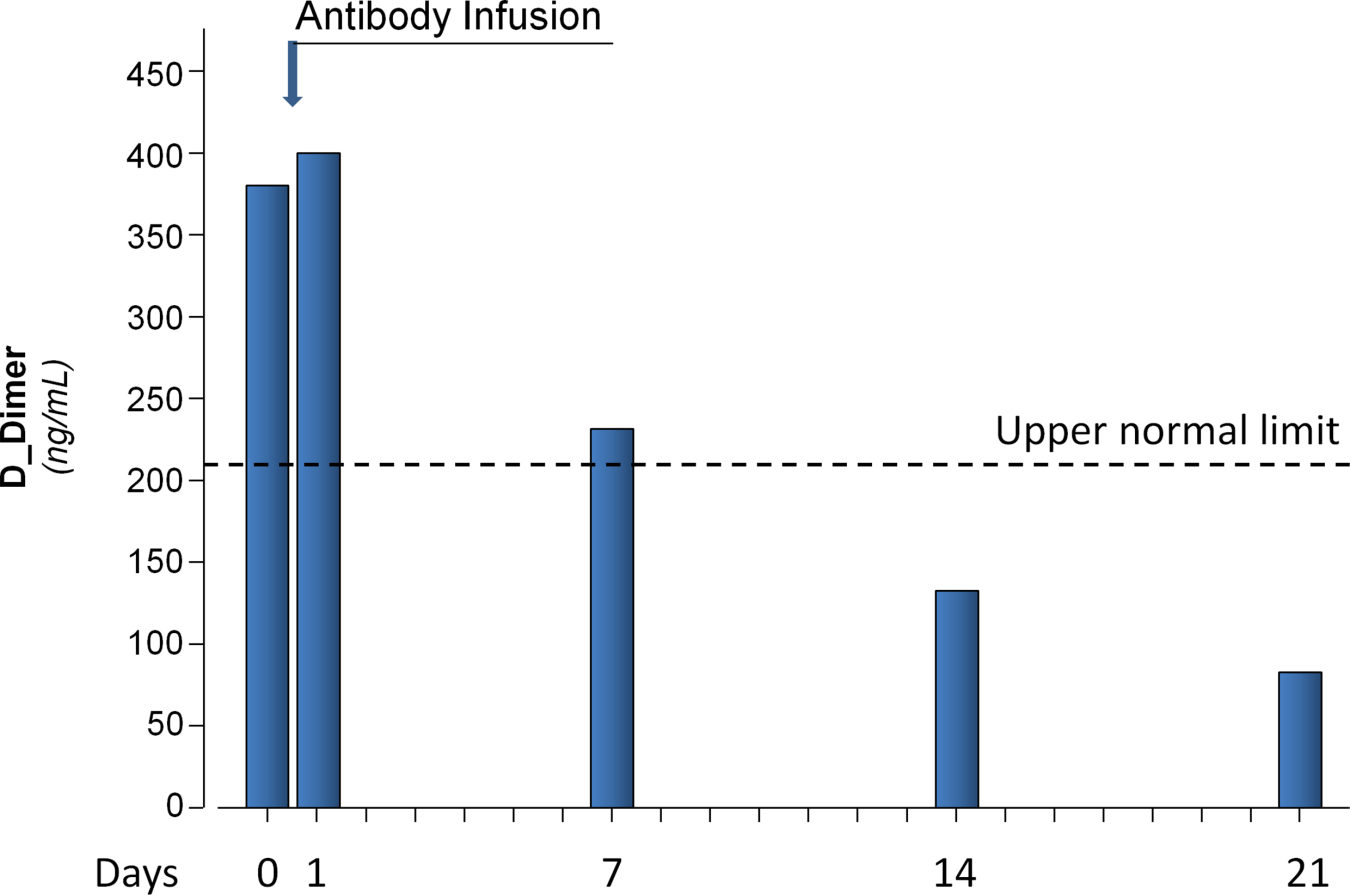
D-Dimer levels in patient plasma samples collected before antibody infusion (Day 0) and 1, 7, 14, and 21 days thereafter. Dashed line represents the upper normal reference value.

On April 6, 2020, a 56-year-old woman was admitted at the Emergency Unit of the Azienda-Socio-Sanitaria-Territoriale (ASST) Papa Giovanni XXIII (Bergamo Hospital, Italy) and then transferred 12 days later to the Intensive Care Unit of the same hospital because of severe respiratory distress secondary to COVID-19 that, after a short unsuccessful trial of Continuous Positive Airways Pressure (CPAP), required intubation for invasive mechanical ventilator support (**Figure 1**). Clinical course was complicated by a urinary tract infection and an Aspergillus super-infection of the lower respiratory tract that fully recovered with specific antimicrobial therapy. Mechanical ventilator support was weaned on May 18 and was followed by a 5-day course of CPAP. Then, she was transferred to the rehabilitation unit on June 8, 2020. However, only 4 days later, on June 12, 2020, she was again admitted to a COVID unit of the same hospital because of cough and worsening dyspnea. At admission, her body temperature was 36.5°C; blood pressure, 98/69 mmHg; heart rate, 98 beats per minute; respiratory rate, 28 breaths per minute; partial artery CO_2_ pressure (PaCO_2_), 32 mmHg; and partial artery O_2_ pressure (PaO_2_) in room air, 59 mmHg. PaO_2_ promptly increased to 96 mmHg with 4 L/min oxygen delivery through a low-flow nasal cannula. Diffuse, bilateral crackles could be auscultated at lungs bases. Laboratory workup showed macrocytic hyperchromic anemia (hemoglobin 9.2 g/dl, MCV 99.3 fl MCH 33.2 pg) with increased C Reactive Protein (11.2 mg/dl, reference range, 0–1 mg/dl), but with a procalcitonin level that was in normal range (0.03 ng/ml). Serum protein electrophoresis showed moderately increased alpha-1, alpha-2 globulins, and beta globulins with only 4% of gamma globulins (reference range, 11.1–18.8%). Flow cytometry found only two CD3- CD19+ B cells per µl (reference range, 200–400 cells/µl) in peripheral blood. There were <3.8 AU/ml of anti-SARS-CoV-2 S1/S2 IgG (Liasorin SARS-CoV-2 IgG chemiluminescent assay [CLIA], Diasorin; negative <12 AU/ml) and 0.02 index of anti-SARS-CoV-2 N IgG (Abbott SARS-CoV-2 IgG chemiluminescent microparticle immunoassay [CMIA]; negative < 1.2 index, **Figure 2**). Quantitative real time polymerase chain reaction (qPCR) failed to detect SARS-CoV-2 nucleic acid in two nasopharyngeal swabs, but qPCR detection of SARS-CoV-2 RNA in a bronco-alveolar lavage (BAL) performed on June 14, after transfer to a sub-intensive care unit, confirmed viral invasion of lower respiratory tract (**Figure 1**). Blood and urinary cultures and extensive laboratory workup for concomitant viral, bacterial or fungal infection were irrelevant. Trans-thoracic echocardiography excluded valve vegetations. Chest computed tomography (CT) scanning showed ground-glass opacities in the dorsal regions of the upper lobes and the right lower lobe, sub pleural consolidations with air bronchogram in the right lower lobe, diffuse traction bronchiectasis, and reticular pattern interstitial abnormalities. This clinical picture was associated with remarkable increases in serum-induced C5b-9 deposition and thrombi formation on cultured human microvascular endothelial cell (HMEC-1) line (**Figures 3A, B**) (13). Plasma Fibrinogen concentration, von Willebrand Factor (vWF) antigen and activity and D-dimer levels (**Figure 4**) were also remarkably increased.

Patient’s family and remote history was irrelevant. Notably, on April 2019, she had been diagnosed with a double-hit diffuse large B-Cell lymphoma that was successfully treated with the first-line protocol of the German Multicenter Study Group for Adult Acute Lymphoblastic Leukemia (R-GMALL) that included six cycles of chemo-immunotherapy with rituximab 375 mg/m^2^, dexamethasone, vincristine, methotrexate, etoposide, and citarabine followed by two additional doses of Rituximab 375 mg/m^2^ every 21 days after completion of the last cycle. This treatment regimen was completed on December 2019.

### Concentrated IgG Solution Collection and Infusion

Seven hundred milliliter of a saline solution of anti-SARS-CoV-2 N IgG (Index 8.8, **Figure 2**) were obtained (after filter washing at the end of the DFPP session) from a 61-year-old male ABO competent adult donor who, according to the clinical and laboratory criteria issued by Consiglio Superiore di Sanità on February 28, 2020 (14), had definitely recovered from COVID-19. He never received blood component transfusions and had negative acid nucleic testing for HAV, HEV, and B19 Parvovirus. The donor was found to have an adequate titer of anti-SARS-CoV-2 S1/S2 (84 UA/ml) and anti-N IgG (7.74 index, **Figure 2**). He provided written informed consent to donation and to the DFPP procedure. The procured IgG concentrated solution was frozen and stored in order to be slowly thawed at 4°C and infused as soon as a consenting ABO competent potential recipient with severe COVID-19 would have become available. The donation was tested as required by the Italian legislation for plasma intended for clinical transfusion (HIV, HCV, HBV nucleic acid, and serology testing, syphilis).

On June 16, 2020, 82 days after symptoms onset, the identified consenting patient received (in the context of an ongoing study approved by the Local Ethical Committee and registered at NCT04346589) a single intravenous infusion of this high titer (4+) anti-S1/S2 and anti-N IgG-enriched cryosupernatant solution without premedication. Plasma cryoprecipitate, which spontaneously separated from cryosupernatant during the thawing procedure, was discarded to avoid the infusion of pro-thrombotic factors—including fibrin, fibrinogen, von Willebrand factor, and others—to the recipient. The IgG-enriched solution was infused in 4 h without acute reactions or signs of fluid overload or cardiovascular instability.

### Outcome

High titer (IgG 4+) of anti-S1/S2 and anti-N IgG were detected by the chromatographic immunoassay qSars-CoV-2 IgG/IgM Cassette Rapid Test (Cellex) in recipient circulation as early as 24 h after the infusion of the IgG enriched solution. Ten days later, the presence of antiviral immunoglobulins was documented by chemiluminescence immunoassay (CLIA) LIASORIN SARS-CoV-2 S1/S2 IgG test (DiaSorin) and subsequently confirmed by chemiluminescence microparticle immunoassay (CMIA) Alinity SARS-CoV-2 IgG (Abbott) detection of anti-nucleocapsid antigen (N) circulating IgG. Serum levels of anti-SARS-CoV-2 N IgG antibodies, which were almost undetectable before antibody infusion, sharply increased at post-infusion day 1 and then slowly and progressively declined at post-infusion days 7, 14, and 21 (**Figure 2**). However, following the infusion antibody titer largely exceeded the upper limit for test positivity (index 1.2) up to day 21 (**Figure 2**).

The fully exogenous origin of circulating anti SARS-CoV-2 IgG was consistent with failure to detect circulating antiviral IgM antibodies and, even more convincing, by persistent depletion of CD3- CD19^+^ B cells in peripheral blood (3 cells/µl). On June 23, just 7 days after antibody infusion, the patient was transferred to a low intensity care Medicine Unit. Notably, in one BAL specimen collected on July 1, 15 days after antibody infusion, SARS-CoV-2 RNA could not be detected by qPCR. Two additional nasopharyngeal swabs tested after the infusion failed to detect any nucleic viral material.

In parallel with viral clearance, patient’s respiratory distress promptly improved after antibody infusion. Her peripheral oxygen saturation (SpO_2_) in room air increased to 96%. PCR decreased from 11.2 to 0.7 mg/dl. IL-6 titer, after peaking (60.5 pg/ml) 1 day after the infusion, progressively dropped to normal levels (2.64 pg/ml) just 8 days after the infusion. Similarly, plasma fibrinogen concentration, vWF antigen and activity, and D-Dimer levels (**Figure 4**), which were remarkably increased before antibody infusion, slightly increased at post infusion day 1 and then progressively decreased on subsequent visits. In particular, D-dimer levels decreased to normal range at post infusion days 14 and 21 (**Figure 4**). A CT imaging 13 days after the infusion showed a marked decrease of the consolidation areas with unmodified reticular abnormalities and bronchiectasis. Consistently, ex vivo serum induced C5b-9 deposition and thrombi formation on HMEC-1 line, after an initial acute increase the day after antibody infusion, progressively decreased, and eventually normalized (**Figures 3A, B**, respectively).

On July 8, 22 days after antibody infusion, and 104 days after disease symptoms onset, the patient was discharged in good health and without chronic sequelae. At 6 months after discharge, she was symptom-free, and a CT imaging showed an almost complete resolution of diffuse interstitial lung involvement. However, hypogammaglobulinemia (IgG concentration: 400 mg/dl) and lymphocytopenia (lymphocyte count: 900/mm^3^) did not recover. Notably, flow cytometry continued to document B-cell depletion from the circulation (two CD3^−^ CD19^+^ B cells per µl).

## Discussion

In this proof-of-concept case report, we describe the outcome of a heavily immune-suppressed adult woman with severe COVID-19 requiring mechanical ventilator support who fully recovered from pneumonia after the infusion of a concentrated solution of anti-SARS-CoV-2 antibodies obtained from a convalescent donor just by one DFPP session. Her immune-compromised status was confirmed not only by previous long-term history of unremitting COVID-19, but also by concomitant opportunistic fungal infections and urinary tract infections. Moreover, she had severe hypogammaglobulinemia, without evidence of circulating anti-SARS-CoV-2 antibodies, and complete depletion of circulating CD19 B cell lymphocytes. Pre-infusion detection of SARS-CoV-2 RNA in a bronco-alveolar lavage confirmed viral invasion of lower respiratory tract up to 80 days after symptoms onset. Thus, we considered that the patient was unable to mount any anti–SARS-CoV-2 humoral response because, in addition to severe immunosuppression related to previous chemotherapy, her CD19^+^ B lymphocytes had been fully and persistently depleted from previous rituximab therapy and, therefore, could not proliferate and mature into antibody-producing plasma cells. Thus, she was an ideal candidate to “passive antibody therapy” (1). Convalescent antibodies were expected to help her immune system by hampering viral engagement with angiotensin receptor enzyme (ACE)-2 receptors expressed on the membrane of target cells, such as alveolar and endothelial cells and many others (15).

Notably, we infused only the cryosupernatant of the concentrate antibody solution and discarded the cryoprecipitate in order not to fuel the pro-thrombotic *milieu* characteristic of more severe cases of COVID-19, with the infusion of fibrin, fibrinogen, von Willebrand factor, and other pro-thrombotic agents (16–19). The appropriateness of this approach was corroborated by finding that biomarkers of inflammation, endothelial activation and hyper-coagulability, such as plasma fibrinogen levels, vWF antigen and activity, and D-Dimer levels, were all increased in the recipient circulation before antibody infusion. Consistently, the infusion was uneventful and was followed by a prompt recovery of the patient who was discharged 22 days later. Notably, the immediate post-infusion appearance of anti-S1/S2 and anti-N IgG immunoglobulins was associated with disappearance of SARS-CoV-2 nucleic material from post infusion bronco-alveolar lavage, a finding consistent with prompt viral clearance from the recipient lungs. In addition, a progressive amelioration of her increased inflammation, endothelial activation and prothrombotic state was highlighted by post-infusion progressive reduction, up to normal range, of plasma fibrinogen levels, von Willebrand factor antigen levels and activity, plasma D-dimer concentration, and ex vivo thrombi formation on HMEC-1 line, respectively. Finding that at recovery visit, 6 months after discharge, CD19^+^ B lymphocytes were persistently depleted from the circulation demonstrated that the immune system of the patient was still unable to sustain any humoral response against the virus.

Our present case, similar to historical COVID-19 patients admitted to the Pneumology Unit of the Bergamo Hospital (Ruggenenti P, PLoS One 2021, submitted), presented with massive terminal complement activation along with ex vivo evidence of increased thrombogenesis. However, ex vivo serum induced C5b-9 and thrombi endothelial deposition, after an immediate acute increase most likely induced by infused antibody binding to SARS-CoV-2, promptly and progressively decreased in our patient after antibody infusion and in parallel with clinical improvement. Conversely, terminal complement activation and thrombophilia persisted in our previous patients (Ruggenenti P, PLoS One 2021, submitted) up to two weeks after initiation of CPAP support and normalized only at post discharge recovery visits, 1 to 2 months after hospital discharge. Thus terminal complement activation and hypercoagulability appeared to parallel disease severity and their improvement appeared to accompany disease recovery. Whether clinical and laboratory changes were two concomitant epiphenomena of the same event (antibody-induced viral clearance) or whether amelioration of complement activation and thrombophilia could be causally related to amelioration of the clinical syndrome merits further investigation.

Several factors may have contributed to the remarkably good outcome of our patient. First, the amount of anti-SARS-CoV-2 antibodies supplied by one concentrated solution obtained by one single DFPP session exceeds the amount supplied by one unit of convalescent plasma (personal communication provided by the supplier of the DFPP device). Second, we infused only the cryosupernatant fraction of our antibody solution. Third, the large majority of patients with severe COVID-19 have unimpaired immune system and may mount a physiological (and in some circumstances even excessive) immune (including humoral) response to the viral aggression. Conceivably, in these circumstances it is difficult to demonstrate an incremental benefit of exogenous antibodies added-on the endogenous physiological immune response. Conversely, as in our patient, it is easier to document a treatment effect when antibody recipient is immunodepressed. This hypothesis is corroborated by recent evidence that convalescent plasma achieved disease remission in a COVID-19 patient unable to produce anti SARS-CoV-2 antibodies because of a common variable immunodeficiency disorder (CVID) (20). This could also explain why prospective randomized trials of convalescent plasma failed to detect any appreciable treatment effect (8) even when antibodies were infused early in the course of viral infection (9) and could be taken to suggest that future trials should probably focus on patients with congenital (20) or acquired (iatrogenic) inability to produce antibodies. In this context, convalescent antibodies could improve disease outcome not only by inhibiting SARS-CoV-2 activity, but also by binding the free S1 spike protein that can directly interact with endothelial cells through the ACE2 receptor and induce endothelial activation with consequent loss of the physiological vascular thromboresistance (21, 22).

Our considerations must be taken with caution because they are based on outcome data of one single, albeit paradigmatic, patient. However, clinical outcome characterized by 82 days of severe, unremitting disease followed by prompt recovery resulting in patient discharge 22 days after antibody infusion, was strongly suggestive of a specific, beneficial effect of passive antibody immunization. Actually, it is very unlikely that specific interventions, different from antibody infusion, might explain the sudden change in patients clinical trajectory.

In conclusion, in this proof of concept case report, we observed that infusion of neutralizing anti-SARS-CoV-2 antibodies induced disease recovery in a severely immunodepressed COVID-19 patient unable to mount any humoral response against the viral aggression because of previous anticancer therapy including repeated courses of rituximab. The observation that passive antibody therapy might prove particularly effective in immunodepressed COVID-19 patients requires evaluation in prospective randomized controlled trials.

## Data Availability Statement

The raw data supporting the conclusions of this article will be made available by the authors, without undue reservation.

## Ethics Statement

The studies involving human participants were reviewed and approved by Azienda Socio Sanitaria Territoriale (ASST) Papa Giovanni XXIII. The patients/participants provided their written informed consent to participate in this study.

## Author Contributions

PR and GR had the original idea. PR wrote the first draft and the final version of the manuscript. DC performed the DFPP procedure and managed the antibody donor. FT collected all clinical and laboratory data. SG, MN, and MG performed the complement tests. AF and MM evaluated, handled and stored the antibody solution and performed the hemostatic tests and anti-SARS-CoV-2 antibody determinations in donor and patients specimens. FR and LL supervised the antibody infusion and managed the recipient throughout the whole hospitalization period. All authors contributed to the article and approved the submitted version.

## Funding

Brembo SpA (Curno, Bergamo, Italy) partially covered study costs by a liberal grant under the initiative “Progetto TrexUno.”

## Conflict of Interest

The authors declare that the research was conducted in the absence of any commercial or financial relationships that could be construed as a potential conflict of interest.
